# Demographic differences in emotional intelligence and facet-level associations with interpersonal relationships among university teachers in Shandong private universities

**DOI:** 10.3389/fpsyg.2026.1790694

**Published:** 2026-05-07

**Authors:** Qianqian Sun, Fang Chen, Luman Wang, Mingcong Sun, Mohd Khairuddin Abdullah

**Affiliations:** 1 Hengxing University of Science and Technology, Qingdao, Shandong, China; 2 Faculty of Psychology and Education, Universiti Malaysia Sabah, Kota Kinabalu, Malaysia; 3 Zhongke Shiyuan New Materials Technology (Qingdao) Co., Ltd., Qingdao, China; 4 Faculty of Education and Sports Studies, Universiti Malaysia Sabah, Kota Kinabalu, Malaysia

**Keywords:** China, emotional intelligence, emotional intelligence facets, faculty development, interpersonal relationships, private higher education, university teachers, workplace relationships

## Abstract

Emotional intelligence (EI) is widely considered a core personal resource for social functioning in educational workplaces, yet evidence remains limited regarding (a) how EI varies across key demographic and work-role characteristics and (b) which EI facets are uniquely associated with workplace interpersonal relationships in private higher education contexts. This study examined EI levels, demographic differences, and face *t*-level associations with interpersonal relationships among university teachers in Shandong private universities in China. Using a two-stage probability sampling design, survey data were collected from 637 teachers. EI was assessed with a 35-item, five-facet scale (self-awareness, managing emotions, motivating oneself, empathy, and social skill), and interpersonal relationships were measured with a 12-item, four-dimension workplace scale (interpersonal regard, mutual acceptance and facilitation, mutual trust, and workplace harmony), both rated on five-point Likert scales. Overall EI was at a moderate level. Group comparisons showed significant EI differences by gender, age, education, professional title, job role, teaching discipline, and administrative/part-time duty experience, whereas teaching years showed no significant differences. EI was positively associated with interpersonal relationships (*r* = 0.570, *p* < 0.01). In multiple regression, the five EI facets jointly accounted for 35.3% of the variance in interpersonal relationships (*R*^2^ = 0.353), with empathy, social skill, and managing emotions showing significant unique associations, while self-awareness and motivating oneself were not significant. These findings map the demographic distribution of EI in China’s private-university teaching workforce and suggest that interpersonal-facing EI facets may represent particularly relevant targets for relationship-focused faculty development.

## Highlights

1. Mapped the distribution of teachers’ emotional intelligence across demographic and work-role strata in Chinese private universities.
2. Demonstrated that emotional intelligence is positively related to collegial relationship quality (respect, acceptance, trust, harmony).
3. Identified interpersonal-facing facets (empathy, social skill, emotion management) as the strongest unique correlates of relationships.
4. Findings support targeted socio-emotional faculty development rather than one-size-fits-all training.

## 1 Introduction

Teachers’ work in contemporary higher education is increasingly characterized by intensified emotional demands and complex interpersonal interactions. Beyond teaching and research, university faculty frequently engage in advising, service, student support, and institutionally assigned tasks that require sustained coordination with colleagues and administrators. In such settings, teachers’ socio-emotional functioning and the quality of workplace relationships are not peripheral concerns; rather, they shape daily cooperation, reduce friction in collaborative work, and support professional functioning under pressure (Bakker and Demerouti, 2017; Demerouti et al., 2001; Hobfoll et al., 2018). These relational processes may be especially salient in private universities, where performance pressures, role overload, and organizational constraints can heighten both the costs of interpersonal friction and the value of collegial support.

Emotional intelligence (EI) is commonly conceptualized as a set of emotion-related competencies that enable individuals to perceive, understand, and regulate emotions effectively in themselves and others. Across education and occupational research, higher EI has consistently been associated with more adaptive psychological functioning and work-related outcomes. In teacher samples, higher EI has been linked to lower burnout risk and more positive occupational adjustment, suggesting that EI may function as a protective personal resource in emotionally demanding professional contexts (Mérida-López and Extremera, 2017; Fiorilli et al., 2019; Geraci et al., 2023). In the broader workplace literature, meta-analytic evidence also indicates that EI is positively associated with job performance and favorable work attitudes, supporting the view that EI is relevant to effective functioning in real-world organizational environments (Joseph and Newman, 2010; O'Boyle et al., 2011; Miao et al., 2017).

A particularly relevant domain in which EI may be linked to workplace functioning is interpersonal relationships (IR), defined here as the quality of collegial interactions reflected in respect, acceptance and supportive exchanges, mutual trust, and workplace harmony. In higher education organizations, faculty members depend on colleagues for informal information sharing, coordination around teaching and service tasks, and relational support in managing routine challenges. Accordingly, IR can be conceptualized as a critical social resource that sustains day-to-day collaboration and may reduce the relational strain associated with high job demands.

The job demands–resources (JD–R) framework and conservation of resources (COR) theory provide a useful basis for understanding why EI may be associated with workplace IR. JD–R theory suggests that personal resources help individuals cope more effectively with job demands and maintain functioning in challenging work environments (Bakker and Demerouti, 2017; Demerouti et al., 2001). COR theory similarly proposes that individuals seek to obtain, preserve, and build valued resources, and that existing resources can facilitate the development of additional resources, including social and relational resources (Hobfoll et al., 2018). From this perspective, EI may be associated with more constructive relationship building by enabling better emotion regulation in conflict situations, more accurate interpretation of colleagues’ emotional cues, and more adaptive communication behaviors. In resource-based terms, EI may not only support individual adjustment but may also help faculty members accumulate and maintain social resources embedded in collegial interactions.

These theoretical perspectives also suggest that EI facets may not be equally relevant to workplace IR. Facets that are more directly enacted in interpersonal contexts, such as empathy and social skill, may be more proximally associated with relationship quality because they involve perspective-taking, emotional attunement, communication, and coordination in interaction. Managing emotions may likewise be especially relevant because it can help individuals respond constructively under stress, ambiguity, or interpersonal tension. By contrast, more intrapersonal facets such as self-awareness and motivating oneself may still matter, but their links to IR may be more indirect and may operate through other interpersonal processes rather than through immediate relational enactment. In this sense, JD–R and COR together imply that different EI facets may vary in their relational relevance, especially in contexts where social coordination is necessary for coping with demanding work conditions.

This issue may be particularly important in private higher education. Private universities often operate under market-oriented pressures, resource constraints, and strong performance expectations, which may intensify teachers’ emotional and relational demands. In such settings, the resource value of EI may become more salient because teachers must not only manage their own emotions but also sustain effective collegial ties under pressure. COR theory further suggests that when organizational demands are heightened, personal resources may become especially important for protecting existing social resources and for building new ones. Thus, the private-university context provides a meaningful setting for examining how different EI facets are associated with workplace IR.

Despite these theoretical expectations, empirical evidence remains uneven in higher education contexts, especially regarding two issues. First, while EI has been widely studied in school and corporate settings, fewer studies have focused on university teachers’ workplace relationships as a core outcome. This is a meaningful gap because collegial relationships in universities support task interdependence, professional learning, and daily coordination, and may therefore represent a key social resource for sustaining performance and wellbeing (Bakker and Demerouti, 2017; Demerouti et al., 2001; Hobfoll et al., 2018). Second, although EI is multidimensional, studies often rely on global EI scores and do not examine whether particular EI facets are more closely associated with workplace IR than others (Joseph and Newman, 2010; Miao et al., 2017; O'Boyle et al., 2011). A third practical gap is that EI may not be evenly distributed across demographic and work-role subgroups; mapping such variation is important for designing targeted faculty development rather than one-size-fits-all training.

This study focuses on full-time teachers from multiple private undergraduate universities in Shandong Province, China. Private higher education constitutes an important part of the broader higher education landscape and serves a substantial number of students, making teacher functioning and workplace climate in this sector practically consequential. By focusing on this context, the study provides evidence that is both theoretically informative and practically relevant for faculty development in demanding higher education environments.

Accordingly, the present study pursues three connected aims aligned with a descriptive-to-associational analytic sequence. First, it describes the overall level and facet profile of EI among teachers in private undergraduate universities. Second, it examines whether overall EI differs across key demographic and work-role variables. Third, it tests (a) the bivariate associations between EI (overall and facets) and workplace IR (overall and dimensions), and (b) the differential patterns of association between EI facets and workplace IR using multiple regression. By clarifying both the distribution of EI and its relational correlates in this context, the study provides evidence relevant to faculty development strategies that integrate socio-emotional competence with relationship-building capacities.

To operationalize these aims, we address the following research questions (RQs):

*RQ_1_*: Do teachers’ EI levels differ across key demographic and work-role characteristics?

*RQ_2_*: Is overall EI positively associated with workplace interpersonal relationships (overall and by dimensions)?

*RQ_3_*: Which EI facets are uniquely associated with workplace interpersonal relationships when modeled simultaneously?

Accordingly, three hypotheses were tested:

*H_1_*: Overall EI differs across demographic and work-role subgroups.

*H_2_*: Overall EI is positively associated with workplace interpersonal relationships.

*H_3_*: EI facets show differential patterns of association with workplace interpersonal relationships when modeled simultaneously, with empathy, social skill, and managing emotions expected to show stronger unique associations than self-awareness and motivating oneself.

## 2 Materials and methods

### 2.1 Study design and setting

This study employed a cross-sectional survey design to examine emotional intelligence (EI), demographic differences in EI, and facet-level associations with workplace interpersonal relationships among university teachers in private universities in Shandong Province, China.

The study focused on full-time teachers working in multiple private undergraduate universities, a context in which collegial interaction and coordination constitute an important component of routine academic work.

### 2.2 Sampling, participants, and data screening

A two-stage probability sampling design was used to enhance both institutional diversity and demographic representativeness. In Stage 1, private undergraduate universities in Shandong Province were selected using region-stratified cluster sampling across major geographic regions. A total of 12 universities were included to ensure institutional and regional diversity. In Stage 2, teachers were sampled within selected universities using stratified random sampling based on gender and teaching experience to ensure representation across key demographic groups.

Data were collected using a structured questionnaire administered online and/or in paper format with institutional support from participating universities. A total of 700 questionnaires were returned. To improve data quality, responses completed in less than 300 s (approximately 6.4 s per item based on 47 total items) and cases showing straightlining patterns (i.e., selecting the same response option across an entire scale) were excluded. The 300-s threshold was adopted because such completion times were considered insufficient to ensure careful reading and cognitive processing of all items and may reflect careless responding (Ward and Meade, 2023). Overall, 63 responses were excluded based on the response-time and straightlining criteria, resulting in a final sample of *N* = 637 and a valid response rate of 91.0%.

The final analytic sample comprised 637 teachers (male = 377, 59.2%; female = 260, 40.8%). Additional participant characteristics are summarized in Table 1.

**Table 1:** Sample characteristics (*N* = 637).

Variable	Category	*n*	%
Gender	Male	377	59.20%
	Female	260	40.80%
Age (years)	≤30	104	16.30%
	31–35	194	30.50%
	36–40	137	21.50%
	41–45	41	6.40%
	46–50	46	7.20%
	≥50	115	18.10%
Teaching years	1–5	211	33.10%
	6–10	174	27.30%
	11–15	144	22.60%
	16–20	62	9.80%
	≥20	46	7.20%
Education	Bachelor’s	16	2.50%
	Master’s	229	35.90%
	Doctoral	392	61.60%
Professional title	Full professor	107	16.80%
	Associate senior	152	23.90%
	Intermediate	199	31.20%
	Junior	179	28.10%
Job role	Frontline teacher	262	41.20%
	Counselor	185	29.10%
	Teaching-research office director	85	13.30%
	Vice dean	78	12.20%
	Dean	27	4.20%
Teaching discipline	Engineering	146	22.90%
	Science	110	17.30%
	Agriculture	71	11.10%
	Medicine	62	9.70%
	Literature	30	4.70%
	Management	82	12.90%
	Arts	57	8.90%
	Education	79	12.50%
Administrative/part-time duty experience	Yes	482	75.70%
	No	155	24.30%

### 2.3 Measures

All constructs were measured using five-point Likert scales (1 = strongly disagree, 5 = strongly agree). Scale structure and internal consistency for the present sample are reported in Table 2. Higher scores indicate higher levels of the corresponding construct.

**Table 2:** Measures and internal consistency.

Construct	Dimension	Items	Cronbach’s α
Emotional intelligence (EI)	Self-awareness (SA)	7	0.916
	Managing emotions (ME)	8	0.896
	Motivating oneself (MO)	6	0.929
	Empathy (EM)	7	0.900
	Social skill (SS)	7	0.914
	Total EI	35	0.945
Workplace interpersonal relationships (IR)	Interpersonal regard (IPR)	3	0.802
	Mutual acceptance and facilitation (MAU)	3	0.845
	Mutual trust (MUT)	3	0.848
	Workplace harmony (WPH)	3	0.840
	Total IR	12	0.883

#### 2.3.1 Emotional intelligence (EI)

EI was assessed using the Leadership Toolkit (2021) Emotional Intelligence Scale, which was psychometrically validated among teachers in Chinese private universities (Sun et al., 2026). The scale comprised 35 items across five facets: self-awareness (SA; 7 items), managing emotions (ME; 8 items), motivating oneself (MO; 6 items), empathy (EM; 7 items), and social skill (SS; 7 items). Internal consistency was high for the total EI scale (Cronbach’s *α* = 0.945) and for each facet (*α* = 0.896–0.929), as shown in Table 2.

#### 2.3.2 Workplace interpersonal relationships (IR)

Workplace interpersonal relationships were measured using a multidimensional workplace interpersonal relationship scale (Singh and Aggarwal, 2022). The final version used in this study included 12 items across four dimensions (3 items each): interpersonal regard (IPR), mutual acceptance and facilitation (MAU), mutual trust (MUT), and workplace harmony (WPH). Internal consistency was good for the total IR scale (Cronbach’s *α* = 0.883) and for each dimension (*α* = 0.802–0.848), as shown in Table 2.

### 2.4 Measurement model evaluation

Confirmatory factor analysis (CFA) was conducted to evaluate the measurement models of EI and IR prior to hypothesis testing. Given that the study involves established reflective constructs, CFA was employed to assess the adequacy of the measurement models and to ensure construct validity (Kline, 2016). Model fit was evaluated using commonly reported indices, including CMIN/DF, RMSEA, SRMR, IFI, TLI, and CFI, following established SEM reporting guidelines (Hu and Bentler, 1999). Convergent validity and construct reliability were evaluated using standardized factor loadings, average variance extracted (AVE), and composite reliability (CR). Discriminant validity was examined by comparing the square roots of AVE with inter-construct correlations (Fornell and Larcker, 1981). CFA fit indices for EI and IR are reported in Table 3.

**Table 3:** Confirmatory factor analysis model fit indices.

Model	CMIN	DF	p	CMIN/DF	RMSEA	SRMR	IFI	TLI	CFI
EI	670.058	550	<0.001	1.218	0.019	0.027	0.991	0.99	0.991
IR	62.688	48	0.076	1.306	0.022	0.019	0.996	0.994	0.996

### 2.5 Common method bias diagnostic

Given the single-source, self-report design, potential common method variance (CMV) was assessed using Harman’s single-factor test as a preliminary diagnostic. All 47 observed items from emotional intelligence and workplace interpersonal relationships were entered simultaneously into an exploratory factor analysis using principal component extraction with no rotation. The first unrotated factor accounted for 31.05% of the total variance, which was below the commonly used 40% threshold (Tang, 2020). In addition, nine factors with eigenvalues greater than 1 were extracted, indicating the absence of a dominant single-factor structure. These results suggest that CMV is unlikely to be a dominant source of variance in the data. However, Harman’s single-factor test cannot rule out CMV entirely, and common method bias cannot be completely excluded in a cross-sectional single-source self-report design. Moreover, common method bias typically inflates correlations among variables. In the present study, however, the facet-level results showed a differentiated pattern, with some emotional intelligence dimensions significantly associated with workplace interpersonal relationships and others not. This pattern suggests that common method bias is unlikely to be the primary driver of the observed associations (Table 4).

**Table 4:** Harman’s single-factor test (summary).

Result	Value	Interpretation
First unrotated factor variance explained	31.05%	Below 40%; no dominant single factor
Number of factors with eigenvalues > 1	9	Multifactor structure supported

### 2.6 Ethical considerations

The studies involving human participants were approved by the Ethics Committee, Faculty of Education and Sport Studies, Universiti Malaysia Sabah. The studies were conducted in accordance with the local legislation and institutional requirements. The participants provided their written informed consent to participate in this study.

Prior to data collection, the researcher communicated with the relevant administrative department (Human Resources Office) of participating universities to explain the study purpose and participation requirements and obtained permission and support. Participation was voluntary. Respondents were informed of anonymity and confidentiality, and the data were used solely for academic research.

### 2.7 Statistical analysis

All tests were two-tailed with *α* = 0.05. Descriptive statistics (mean, standard deviation, skewness, and kurtosis) were computed for EI and its facets. Independent-samples *t*-tests and one-way ANOVA were conducted to examine demographic and work-role differences in EI. Where relevant, homogeneity of variance was examined (e.g., Levene’s test), and appropriate robust procedures were considered when assumptions were violated (e.g., Welch ANOVA and Games–Howell *post hoc* tests). Following significant omnibus ANOVA results, LSD *post hoc* tests were applied to identify pairwise group differences. Pearson correlations were computed to estimate associations between EI (total and facets) and workplace interpersonal relationships. Finally, multiple linear regression was performed to examine facet-level associations with workplace interpersonal relationships, with the five EI facets entered simultaneously as predictors. Multicollinearity was assessed using tolerance and variance inflation factor (VIF). All analyses were conducted using SPSS (version 27.0), and CFA was conducted using AMOS (version 27.0). Where appropriate, effect sizes were reported (e.g., Cohen’s *d* for *t*-tests; *η*^2^/partial *η*^2^ for ANOVA).

## 3 Results

### 3.1 Descriptive statistics of emotional intelligence

Descriptive statistics for EI and its five facets are presented in Table 5. Overall EI was moderate (*M* = 3.552, SD = 0.719). Among the five facets, motivating oneself (MO) showed the highest mean score (*M* = 3.717, SD = 1.069), followed by empathy (EM; *M* = 3.668, SD = 0.933) and managing emotions (ME; *M* = 3.581, SD = 0.878), social skill (SS; *M* = 3.460, SD = 1.007) and self-awareness (SA; *M* = 3.355, SD = 1.033) were comparatively lower. Skewness and kurtosis values suggested no severe non-normality. Minimum and maximum values reflect averaged scale scores rather than single-item responses.

**Table 5:** Descriptive statistics of EI (*N* = 637).

Variable	Min	Max	Mean	SD	Skewness	Kurtosis
Self-awareness (SA)	1.1	4.9	3.355	1.033	−0.775	−0.989
Managing emotions (ME)	1.4	4.8	3.581	0.878	−1.112	−0.244
Motivating oneself (MO)	1.2	5	3.717	1.069	−1.381	0.411
Empathy (EM)	1.1	5	3.668	0.933	−1.197	0.069
Social skill (SS)	1	4.9	3.46	1.007	−1.006	−0.499
Total EI	1.5	4.4	3.552	0.719	−0.939	−0.221

### 3.2 Demographic differences in emotional intelligence

As shown in Table 6, total emotional intelligence (EI) differed significantly across several demographic and work-role characteristics. Female teachers reported higher total EI than male teachers, *t* = −2.36, *p* = 0.019, *d* = 0.18, indicating a small effect. At the facet level, gender differences were concentrated in motivating oneself (MO), empathy (EM), and social skill (SS), with females scoring higher than males on these three facets, whereas self-awareness (SA) and managing emotions (ME) did not differ significantly by gender. Taken together, the gender pattern suggests that overall EI differences were present but were not uniform across all facets.

**Table 6:** Group differences in Emotional Intelligence (EI) and its facets across demographic and work-role variables (*N* = 637).

Variable	Category	Total EI (*M* ± SD)	SA (*M* ± SD)	ME (*M* ± SD)	MO (*M* ± SD)	EM (*M* ± SD)	SS (*M* ± SD)
Gender	Male	3.50 ± 0.76	3.35 ± 1.04	3.55 ± 0.90	3.62 ± 1.13	3.58 ± 0.96	3.39 ± 1.04
	Female	3.63 ± 0.65	3.36 ± 1.02	3.63 ± 0.84	3.85 ± 0.95	3.79 ± 0.88	3.56 ± 0.95
	*t*	−2.36*	−0.041	−1.074	−2.709**	−2.832**	−2.147*
	*P*	0.019	0.967	0.283	0.007	0.005	0.032
	Effect size (Cohen’s *d*)	0.18	0.01	0.09	0.22	0.23	0.17
Age (years)	a. ≤30	3.28 ± 0.79	3.12 ± 1.10	3.29 ± 1.02	3.46 ± 1.19	3.44 ± 1.03	3.13 ± 1.18
	b. 31–35	3.53 ± 0.72	3.22 ± 1.07	3.55 ± 0.88	3.78 ± 1.01	3.67 ± 0.92	3.45 ± 1.02
	c. 36–40	3.56 ± 0.77	3.41 ± 1.02	3.56 ± 0.84	3.73 ± 1.08	3.65 ± 0.99	3.46 ± 1.00
	d. 41–45	3.61 ± 0.68	3.39 ± 0.99	3.64 ± 0.93	3.61 ± 1.12	3.70 ± 0.88	3.70 ± 0.80
	e. 46–50	3.72 ± 0.64	3.64 ± 0.78	3.66 ± 0.86	3.75 ± 1.06	3.87 ± 0.80	3.69 ± 0.82
	f. ≥50	3.75 ± 0.55	3.61 ± 0.96	3.87 ± 0.67	3.86 ± 1.01	3.81 ± 0.82	3.60 ± 0.90
	*F*	5.405***	4.088**	5.122***	1.784	2.229	3.728**
	*P*	<0.001	0.001	<0.001	0.114	0.05	0.002
	LSD-test	f > b,c,d,e; e > a	f,e > a,b; c > a	f > a,b,c; b,c,d,e > a	–	–	f,e,d,c,b > a
	Effect size (partial *η*^2^)	0.041	0.031	0.039	0.014	0.017	0.029
Teaching years	a. 1–5	3.50 ± 0.72	3.31 ± 1.07	3.56 ± 0.90	3.67 ± 1.08	3.58 ± 0.94	3.37 ± 1.08
	b. 6–10	3.56 ± 0.76	3.29 ± 1.03	3.56 ± 0.86	3.82 ± 0.99	3.68 ± 1.00	3.47 ± 0.98
	c. 11–15	3.58 ± 0.70	3.41 ± 0.96	3.57 ± 0.87	3.61 ± 1.14	3.71 ± 0.88	3.61 ± 0.91
	d. 16–20	3.52 ± 0.71	3.31 ± 1.16	3.62 ± 0.95	3.72 ± 1.05	3.60 ± 0.95	3.38 ± 1.00
	e. ≥20	3.75 ± 0.62	3.67 ± 0.86	3.72 ± 0.77	3.87 ± 1.10	4.00 ± 0.70	3.51 ± 1.04
	*F*	1.274	1.467	0.37	1.156	2.083	1.324
	*P*	0.279	0.21	0.83	0.329	0.082	0.26
	LSD-test	–	–	–	–	–	–
	Effect size (partial *η*^2^)	0.008	0.009	0.002	0.007	0.013	0.008
Education	a. Bachelor’s	2.63 ± 0.91	2.28 ± 1.01	2.86 ± 1.02	2.73 ± 1.29	2.88 ± 0.99	2.38 ± 1.02
	b. Master’s	3.55 ± 0.74	3.37 ± 1.03	3.59 ± 0.87	3.66 ± 1.11	3.65 ± 0.95	3.48 ± 1.02
	c. Doctoral	3.59 ± 0.67	3.39 ± 1.02	3.61 ± 0.86	3.79 ± 1.01	3.71 ± 0.91	3.49 ± 0.98
	*F*	14.329***	9.177***	5.669**	8.287***	6.150**	9.644***
	*P*	<0.001	<0.001	0.004	<0.001	0.002	<0.001
	LSD-test	b,c > a					
	Effect size (partial *η*^2^)	0.043	0.028	0.018	0.026	0.019	0.029
Professional title	a. Full professor	3.77 ± 0.51	3.63 ± 0.93	3.94 ± 0.56	3.88 ± 0.94	3.79 ± 0.82	3.58 ± 0.90
	b. Associate senior	3.60 ± 0.73	3.36 ± 0.98	3.55 ± 0.91	3.71 ± 1.12	3.80 ± 0.84	3.59 ± 0.90
	c. Intermediate	3.52 ± 0.75	3.34 ± 1.05	3.54 ± 0.86	3.69 ± 1.09	3.62 ± 0.99	3.45 ± 1.03
	d. Junior	3.42 ± 0.75	3.20 ± 1.09	3.44 ± 0.98	3.65 ± 1.08	3.54 ± 0.99	3.30 ± 1.10
	*F*	5.55**	3.905**	7.724***	1.145	3.133*	2.876*
	*P*	0.001	0.009	<0.001	0.33	0.025	0.035
	LSD-test	a > c,d; b > d	a > b,c,d	a > b,c,d	–	a,b > d	a,b > d
	Effect size (partial η^2^)	0.026	0.018	0.035	0.005	0.015	0.013
Job role	a. Frontline teacher	3.53 ± 0.72	3.40 ± 1.01	3.53 ± 0.88	3.69 ± 1.09	3.61 ± 0.96	3.48 ± 1.01
	b. Counselor	3.47 ± 0.78	3.22 ± 1.08	3.48 ± 0.95	3.67 ± 1.07	3.64 ± 0.97	3.35 ± 1.08
	c. Teaching-research office director	3.55 ± 0.76	3.34 ± 1.02	3.59 ± 0.88	3.63 ± 1.21	3.73 ± 0.90	3.46 ± 0.98
	d. Vice dean	3.75 ± 0.51	3.53 ± 1.03	3.91 ± 0.58	3.92 ± 0.90	3.74 ± 0.87	3.67 ± 0.87
	e. Dean	3.73 ± 0.52	3.38 ± 0.93	3.80 ± 0.85	4.01 ± 0.74	4.06 ± 0.50	3.45 ± 0.86
	*F*	2.620*	1.415	3.928**	1.445	1.745	1.405
	*P*	0.034	0.228	0.004	0.218	0.138	0.231
	LSD-test	d > a,b	–	d > a,b,c	–	–	–
	Effect size (partial *η*^2^)	0.016	0.009	0.024	0.009	0.011	0.009
Teaching discipline	a. Engineering	3.44 ± 0.75	3.22 ± 1.10	3.51 ± 0.88	3.56 ± 1.14	3.55 ± 1.00	3.38 ± 1.01
	b. Science	3.66 ± 0.71	3.49 ± 0.94	3.64 ± 0.84	3.93 ± 0.92	3.67 ± 0.96	3.60 ± 0.95
	c. Agriculture	3.39 ± 0.77	3.33 ± 1.02	3.47 ± 0.97	3.59 ± 1.17	3.36 ± 1.11	3.21 ± 1.21
	d. Medicine	3.50 ± 0.69	3.25 ± 1.07	3.45 ± 0.95	3.71 ± 1.05	3.70 ± 0.89	3.42 ± 1.01
	e. Literature	3.59 ± 0.59	3.71 ± 0.72	3.58 ± 0.80	3.49 ± 1.10	3.65 ± 0.87	3.53 ± 0.97
	f. Management	3.50 ± 0.77	3.22 ± 1.13	3.57 ± 0.88	3.70 ± 1.11	3.71 ± 0.87	3.29 ± 1.09
	g. Arts	3.74 ± 0.60	3.43 ± 1.00	3.76 ± 0.84	3.79 ± 1.05	4.01 ± 0.62	3.72 ± 0.85
	h. Education	3.70 ± 0.66	3.46 ± 1.00	3.70 ± 0.82	3.87 ± 0.96	3.86 ± 0.82	3.63 ± 0.83
	*F*	2.575*	1.629	1.089	1.725	3.029**	2.367*
	*P*	0.013	0.124	0.369	0.1	0.004	0.022
	LSD-test	b > a,c; g > a,c,f; h > a,c	–	–	–	h > c,f; g > a,c,f	b,h > c,f; g > a,c,f
	Effect size (partial *η*^2^)	0.028	0.018	0.012	0.019	0.033	0.026
Administrative/part-time duty experience	Yes	3.60 ± 0.69	3.40 ± 1.01	3.64 ± 0.84	3.78 ± 1.01	3.72 ± 0.92	3.50 ± 1.00
	No	3.40 ± 0.79	3.22 ± 1.08	3.41 ± 0.96	3.52 ± 1.21	3.52 ± 0.97	3.35 ± 1.03
	*t*	2.818**	1.862	2.589*	2.373*	2.228*	1.541
	*P*	0.005	0.064	0.01	0.018	0.027	0.124
	Effect size (Cohen’s *d*)	0.28	0.18	0.26	0.24	0.21	0.15

Values are mean ± SD. SA, self-awareness; ME, managing emotions; MO, motivating oneself; EM, empathy; SS, social skill. Post hoc comparisons were conducted using Fisher’s LSD following significant omnibus ANOVA results. For two-group comparisons, Cohen’s d is reported as the effect size; for multi-group comparisons, partial *η*^2^ is reported. Conventional benchmarks are approximately 0.20/0.50/0.80 for d and 0.01/0.06/0.14 for partial *η*^2^. ***p < 0.001, **p < 0.01, *p < 0.05.

Age groups also showed significant differences in total EI, *F*(5, 631) = 5.405, *p* < 0.001, partial *η*^2^ = 0.041, indicating a small effect. Mean scores indicated an overall age-related gradient, with the highest total EI observed in the ≥50 group and the lowest in the ≤30 group. *Post hoc* comparisons suggested that the oldest group scored higher than several younger and mid-age groups, and the 46–50 group scored higher than the youngest group. At the facet level, significant age-group differences emerged for SA, ME, and SS, whereas MO did not vary significantly across age groups. For EM, the omnibus test was marginal, *F*(5, 631) = 2.229, *p* = 0.050, and is therefore interpreted cautiously. Overall, these findings suggest that age was associated with several dimensions of EI, especially those more closely related to self-regulation and social functioning.

Teaching years did not significantly differentiate total EI, *F*(4, 632) = 1.274, *p* = 0.279, partial *η*^2^ = 0.008, and no significant differences were observed for any EI facet. This indicates that length of teaching experience alone was not associated with overall EI levels in this sample. One possible explanation is that total teaching years may not fully capture institution-specific role exposure or organizational embeddedness.

Education level was strongly associated with total EI, *F*(2, 634) = 14.329, *p* < 0.001, partial *η*^2^ = 0.043, indicating a small effect. Teachers with doctoral and master’s degrees reported higher total EI than those with a bachelor’s degree, and the same general pattern was observed across all five EI facets. *Post hoc* comparisons confirmed that both the master’s and doctoral groups scored higher than the bachelor’s group. This suggests that educational attainment was one of the most consistent demographic correlates of EI in the present sample.

Professional title also showed significant differences in total EI, *F*(3, 633) = 5.550, *p* = 0.001, partial *η*^2^ = 0.026, indicating a small effect. Full professors reported the highest total EI, and *post hoc* results indicated that they scored higher than teachers with intermediate and junior titles, while associate senior teachers scored higher than junior teachers. Facet-level analyses showed significant differences for SA, ME, EM, and SS, whereas MO did not vary significantly by title. Taken together, these results suggest a relatively clear title-related gradient in EI, particularly for facets linked to regulation and interpersonal functioning.

Job role was related to total EI, *F*(4, 632) = 2.620, *p* = 0.034, partial *η*^2^ = 0.016, indicating a small effect. Administrators, especially vice deans, showed higher mean EI scores than frontline teachers and counselors. Among the five facets, only ME differed significantly across job roles, again favoring administrative positions over non-administrative roles. This pattern suggests that role complexity and administrative responsibilities may be more closely linked to emotional regulation than to all aspects of EI more broadly.

Teaching discipline was also associated with total EI, *F*(7, 629) = 2.575, *p* = 0.013, partial *η*^2^ = 0.028, indicating a small effect. Arts and Education showed the highest mean total EI, whereas Agriculture showed the lowest. Discipline differences were facet-specific and reached significance for EM and SS, with *post hoc* comparisons indicating relatively higher scores in Arts and Education than in several other disciplines. Overall, the disciplinary pattern suggests that EI differences across subject areas were present, but were more evident for interpersonal rather than purely intrapersonal facets.

Finally, administrative/part-time duty experience was associated with higher total EI, *t* = 2.818, *p* = 0.005, *d* = 0.28, indicating a small effect. Teachers who had undertaken institutionally assigned administrative or other part-time duties reported higher total EI than those without such experience. This pattern was also observed for ME, MO, and EM, whereas differences in SA and SS were not statistically significant. Taken together, these findings suggest that extra-role experience was associated with higher EI overall, particularly in facets related to motivation, emotional regulation, and empathy.

Overall, these subgroup analyses indicate that total EI varied across most demographic and work-role characteristics, including gender, age, education, professional title, job role, teaching discipline, and administrative/part-time duty experience, whereas teaching years showed no significant differences. Thus, H_1_ was broadly supported, with the exception of teaching years, and several observed differences were facet-specific.

### 3.3 Correlation analysis of emotional intelligence and workplace interpersonal relationships

Table 7 presents the correlations among total emotional intelligence (EI), its five facets, and workplace interpersonal relationships (IR). Overall EI was positively associated with total IR (*r* = 0.570, *p* < 0.01), indicating a moderate positive relationship between teachers’ emotional intelligence and the quality of workplace interpersonal relationships. In addition, total EI was significantly and positively associated with each IR dimension, including interpersonal regard (IPR), mutual acceptance and facilitation (MAU), mutual trust (MUT), and workplace harmony (WPH), suggesting that higher EI was associated with more positive patterns of collegial interaction across multiple relational domains.

**Table 7:** Pearson correlations between EI (total/facets) and workplace interpersonal relationships (IR: total/dimensions) (*N* = 637).

Variable	IR	IPR	MAU	MUT	WPH	EI	SA	ME	MO	EM	SS
IR	1										
IPR	0.719**	1									
MAU	0.762**	0.417**	1								
MUT	0.773**	0.424**	0.412**	1							
WPH	0.794**	0.415**	0.494**	0.487**	1						
EI	0.570**	0.412**	0.383**	0.506**	0.433**	1					
SA	0.368**	0.269**	0.252**	0.332**	0.267**	0.776**	1				
ME	0.437**	0.329**	0.312**	0.346**	0.344**	0.735**	0.510**	1			
MO	0.355**	0.221**	0.244**	0.337**	0.271**	0.708**	0.437**	0.369**	1		
EM	0.493**	0.388**	0.302**	0.445**	0.367**	0.716**	0.403**	0.418**	0.414**	1	
SS	0.444**	0.308**	0.297**	0.400**	0.343**	0.736**	0.466**	0.380**	0.416**	0.423**	1

**p < 0.01 (two-tailed).

At the facet level, all five EI dimensions were significantly and positively associated with total IR, although the magnitudes of these correlations varied. Empathy (EM), social skill (SS), and managing emotions (ME) showed comparatively stronger associations with total IR and its dimensions, whereas self-awareness (SA) and motivating oneself (MO) showed relatively weaker, though still significant, correlations. This pattern suggests that both intrapersonal and interpersonal components of EI were relevant to workplace IR, but that facets more directly enacted in social interaction may have shown stronger associations with relationship quality.

Correlations among the five EI facets ranged from 0.369 to 0.510, indicating moderate intercorrelations rather than excessive overlap. This pattern supports the decision to examine EI at the facet level, as the five dimensions appear to be related yet sufficiently distinct to justify separate analysis. Taken together, these correlation results provide initial support for H_2_ and indicate that EI was meaningfully associated with workplace interpersonal relationships in this sample.

### 3.4 Multiple regression analysis of facet-level associations with workplace interpersonal relationships

To examine the unique associations between EI facets and workplace interpersonal relationships, the five EI dimensions were entered simultaneously into a multiple regression model predicting total IR. As shown in Table 8, the overall model was statistically significant and accounted for 35.3% of the variance in workplace interpersonal relationships (*R*^2^ = 0.353, *F* = 68.837, *p* < 0.001), indicating that the five EI facets jointly accounted for a meaningful proportion of variance in IR in this sample.

**Table 8:** Multiple regression predicting workplace interpersonal relationships from EI facets (*N* = 637).

Predictor	*B*	Std. error	*β*	*t*	*p*	Tolerance	VIF
Constant	1.415	0.129	–	10.974	<0.001	–	–
Self-awareness (SA)	0.019	0.031	0.025	0.613	0.540	0.613	1.632
Managing emotions (ME)	0.180	0.035	0.203	5.173	<0.001	0.669	1.494
Motivating oneself (MO)	0.047	0.028	0.065	1.699	0.090	0.704	1.420
Empathy (EM)	0.236	0.032	0.283	7.366	<0.001	0.696	1.436
Social skill (SS)	0.161	0.030	0.209	5.381	<0.001	0.682	1.466

B = unstandardized coefficients; β = standardized coefficients. Model fit: *R* = 0.594; *R*^2^ = 0.353; *F* = 68.733; *p* < 0.001. VIF = 1.420–1.632 (<5), indicating no serious multicollinearity (Daoud, 2017; Hair et al., 2019). *p < 0.05, *p* < 0.01, *p < 0.001.

At the facet level, empathy (EM), social skill (SS), and managing emotions (ME) were significantly associated with workplace interpersonal relationships, whereas self-awareness (SA) and motivating oneself (MO) were not significant when all five facets were modeled simultaneously. Among the significant predictors, empathy showed the strongest association, followed by social skill and managing emotions. This pattern suggests that EI facets more directly enacted in interpersonal interaction and emotion regulation may be more proximally associated with workplace relationship quality than facets that are primarily intrapersonal in nature.

The collinearity diagnostics were satisfactory, with tolerance values ranging from 0.613 to 0.704 and VIF values ranging from 1.420 to 1.632, indicating that multicollinearity was not a concern and that the five EI facets were sufficiently distinct to be examined together in the same model. This result is consistent with the correlation analysis, which showed moderate rather than excessive overlap among the EI dimensions.

Taken together, the regression findings indicate that the associations between EI and workplace IR were not uniform across facets. Rather, the pattern was concentrated in empathy, social skill, and managing emotions, whereas self-awareness and motivating oneself did not show unique associations after shared variance across facets was taken into account. Accordingly, H_3_ was supported, as the observed pattern was consistent with the hypothesized differential facet-level associations.

## 4 Discussion

### 4.1 Summary of key findings

This study examined emotional intelligence (EI) and workplace interpersonal relationships (IR) among teachers in private universities in Shandong Province, China, focusing on (a) the level and profile of EI facets, (b) subgroup differences across demographic and work-role characteristics, and (c) whether EI and its facets are uniquely associated with workplace IR. Three findings are central. First, overall EI was moderate, with relatively higher scores in Motivating oneself and Empathy, and comparatively lower scores in Self-awareness and Social skill. Second, EI differed across multiple demographic/work-role characteristics (gender, age, education, professional title, job role, discipline, and administrative/part-time duty experience), whereas teaching years showed no consistent differentiation. Third, EI was positively associated with workplace IR, and in a facet-level model Managing emotions, Empathy, and Social skill emerged as robust unique correlates of IR, while Self-awareness and Motivating oneself did not retain independent associations once overlap among EI facets was controlled. Collectively, these findings extend the literature on EI and relationship quality into Chinese private higher education, where relational coordination and role hybridity (teaching plus extra-role assignments) are salient features of academic work (Zhang et al., 2021; Wilkins and Neri, 2019).

### 4.2 Interpreting the EI facet profile: capability signals under contextual demands

The observed profile—stronger motivational and empathic tendencies alongside comparatively weaker self-awareness and social skill—can be interpreted as an adaptation to context rather than a simple “strength–weakness” pattern. Private higher education often involves intensified performance management, high student-service expectations, and frequent coordination across units, which may increase emotional labor and reward “getting things done” and staying responsive to others (Wilkins and Neri, 2019). In Chinese private universities, recent evidence further suggests that teachers’ work concerns are closely associated with teaching effectiveness, underscoring the practical relevance of socio-emotional resources that can buffer strain and support professional functioning (Liang et al., 2025). From a job demands–resources perspective, socio-emotional competencies function as personal resources that help employees sustain performance and social functioning under elevated demands (Bakker and Demerouti, 2017; Schaufeli, 2017). Consistent with conservation of resources theory, individuals are motivated to deploy and build resources (e.g., regulation skills, empathic responding) to protect against resource loss and maintain valued outcomes such as social support and trust—especially in demanding environments (Hobfoll et al., 2018; Chen et al., 2019).

At the same time, the relatively lower mean levels of Self-awareness and Social skill should be interpreted dialectically. One interpretation is contextual: self-awareness may be less visible and less institutionally reinforced in routinized, compliance-heavy systems, while social skill (influence, coordination, relationship repair) may depend strongly on psychological safety and collegial openness—conditions that vary widely across departments and institutions (Frazier et al., 2017; Newman et al., 2017; Liu et al., 2016). A competing explanation is measurement-related: self-report EI captures perceived competence and self-concept as much as actual ability, which can be shaped by organizational norms regarding modesty, hierarchy, and “appropriate” self-disclosure (MacCann et al., 2020; Mayer et al., 2016). Thus, the facet profile likely reflects a mixture of contextual activation, institutional reinforcement, and self-perception processes rather than stable deficits. In this sense, the private-university context may heighten the practical relevance of EI because emotionally and relationally demanding work conditions can increase the value of personal resources for sustaining collegial functioning.

### 4.3 Subgroup differences: what they likely mean—and what they may not mean

The subgroup patterns indicate meaningful heterogeneity in EI, but several findings invite a more cautious, critical reading.

Gender. Female teachers reported higher overall EI and higher scores in Motivating oneself, Empathy, and Social skill. This aligns with a broad evidence base showing gender differences in interpersonal sensitivity and relational orientation, which can translate into higher self-reported empathic and social competencies (Hsu et al., 2022; MacCann et al., 2020). However, the literature also suggests that gender gaps are contingent on EI conceptualization (ability vs. trait), measurement format (performance vs. self-report), and cultural context (Mayer et al., 2016; MacCann et al., 2020). Therefore, an alternative explanation is reporting style: women may be more willing to endorse relational self-descriptions, whereas men may underreport such tendencies due to social norms. Multi-method designs (e.g., ability EI, peer ratings) are needed to disentangle substantive versus perceptual effects.

Age versus teaching years. Age gradients were visible for total EI and select facets, whereas teaching years did not differentiate EI. This divergence is theoretically plausible: age reflects broader life-course socio-emotional learning and role exposure beyond teaching tenure, while teaching years—especially in coarse categories—may be confounded by role mobility, institution switching, or uneven access to professional development (Bakker and Demerouti, 2017; Hobfoll et al., 2018). In addition, teaching years may not fully reflect teachers’ institutional tenure within their current universities. Some faculty members may have accumulated substantial teaching experience overall but may still be relatively new to their present institutions, thereby having had fewer opportunities to develop stable collegial ties. A competing explanation is restricted variance: if many participants cluster in mid-career bands, teaching-years effects can attenuate even when age effects remain detectable. This suggests that “experience” should be modeled more precisely (e.g., leadership-role exposure, mentoring experience, critical incidents) rather than years alone.

Education, professional title, job role, and administrative/extra-role experience. Higher EI among doctoral-degree holders, senior titles, administrators, and those with extra-role experience can be interpreted in at least two competing ways. The selection interpretation is that socio-emotionally competent individuals are more likely to attain advanced degrees, be promoted, and be entrusted with institutional duties. The socialization interpretation is that leadership-adjacent roles cultivate emotion regulation, empathic communication, and coordination skills through repeated practice under high-stakes interaction demands (Wenner and Campbell, 2017; Liu et al., 2016; Côté, 2017). Cross-sectional data cannot adjudicate these mechanisms; nevertheless, the pattern is consistent with the idea that relational capability and role complexity are mutually reinforcing in academic organizations (Bakker and Demerouti, 2017; Hobfoll et al., 2018).

### 4.4 EI as a relational resource: why some facets matter more than others

Importantly, this facet-level pattern clarifies why treating EI as a unitary construct may obscure the relational mechanisms most relevant for collegial functioning in higher education. A central contribution of this study is clarifying that EI is positively associated with workplace IR and that EI facets differ in the strength of their unique associations once their shared variance is considered. The positive EI–IR association is consistent with meta-analytic and review evidence that EI supports relationship maintenance, constructive conflict handling, and cooperative coordination—processes that cumulatively shape trust and relational quality at work (Miao et al., 2017; Côté, 2017; MacCann et al., 2020). In educational organizations, relationship quality among colleagues is particularly consequential because improvement work and informal influence often rely on collaboration and peer interaction rather than formal authority (Liu et al., 2016; Wenner and Campbell, 2017).

At the facet level, Managing emotions, Empathy, and Social skill emerged as unique correlates of IR. This pattern is theoretically coherent. Emotion regulation helps teachers remain constructive under interpersonal friction, reduce escalation, and respond flexibly to ambiguity—supporting workplace harmony and trust-building (Hobfoll et al., 2018; Mérida-López and Extremera, 2017; Côté, 2017). Empathy facilitates perspective-taking and prosocial responsiveness, strengthening mutual acceptance and relational safety—key ingredients for collaboration and sustained collegial ties (Miao et al., 2017; MacCann et al., 2020; Mérida-López and Extremera, 2017). Social skill supports effective communication, influence, and coordination routines—central elements of maintaining cooperative networks in organizations (Côté, 2017; Miao et al., 2017; Liu et al., 2016).

Equally important is what did not emerge: Self-awareness and Motivating oneself were not independently associated with IR after controlling other facets. A dialectical interpretation is that these facets may be more distal to relationship quality and operate indirectly through more proximal interpersonal enactment skills. For example, self-awareness may matter insofar as it enables regulation and empathic responding; once regulation and empathy are included, the residual variance in self-awareness may be less relationally consequential. Similarly, self-motivation may support persistence and performance but translate into better relationships only when paired with communication and repair skills (Bakker and Demerouti, 2017; Hobfoll et al., 2018). This distal-to-proximal logic is consistent with resource-based accounts, where intrapersonal resources shape interpersonal outcomes through behavioral channels and context-dependent opportunities (Bakker and Demerouti, 2017; Schaufeli, 2017). This interpretation is consistent with JD–R and COR perspectives in suggesting that personal resources may differ in their relational relevance depending on whether they are directly enacted in social interaction or operate more indirectly through other behavioral and contextual pathways.

### 4.5 Contributions and boundary conditions: what this study adds to the literature

This study contributes to the literature in three ways. First, it provides evidence from Chinese private higher education, a context underrepresented in EI–workplace relationship research relative to K–12 and Western organizational samples, and one characterized by strong service demands and organizational hybridity (Zhang et al., 2021; Wilkins and Neri, 2019). Second, by focusing on facet-level unique associations, it moves beyond treating EI as a unitary construct and identifies relationally proximal competencies most directly linked to workplace IR (MacCann et al., 2020; Miao et al., 2017). Third, the observed subgroup patterns suggest that socio-emotional capabilities may be intertwined with role exposure (administrative/extra-role duties) and organizational positioning (title, job role), pointing to plausible bidirectional dynamics between relational competence and institutional participation (Bakker and Demerouti, 2017; Hobfoll et al., 2018; Liu et al., 2016).

At the same time, the results likely depend on boundary conditions. Relationship quality is not solely an individual attribute; it is also shaped by team norms, psychological safety, leadership practices, and structural constraints. Hence, EI may translate into IR more strongly in climates that reward open communication and provide safe conditions for interpersonal repair (Frazier et al., 2017; Newman et al., 2017; Liu et al., 2016). In more hierarchical or punitive climates, EI may be used defensively or remain underutilized, weakening the observed link. This underscores the need to treat EI as a person–context phenomenon rather than a purely individual trait.

## 5 Conclusion, practical implications, limitations, and future research

### 5.1 Conclusion

This study investigated emotional intelligence (EI), subgroup differences in EI, and the facet-level associations between EI and workplace interpersonal relationships (IR) among teachers in private universities in Shandong Province, China. Three conclusions can be drawn. First, teachers reported a moderate level of EI, with a profile indicating relatively stronger motivational and empathic tendencies and comparatively lower self-awareness and social interaction competence. Second, EI varied across multiple demographic and role-related strata (gender, age, education, professional title, job role, discipline, and administrative/part-time duty experience), whereas teaching years did not show consistent differentiation, suggesting that role exposure and broader life-course development may be more informative than tenure alone. Third, EI was positively associated with workplace IR, and the facet-level model indicated that Managing emotions, Empathy, and Social skill showed the strongest unique associations with relationship quality when controlling for overlap among EI dimensions. Overall, these findings suggest that EI—especially its relationally proximal facets—may represent a meaningful socio-emotional resource for sustaining healthy workplace relationships in relationally intensive academic environments (Miao et al., 2017; Côté, 2017; Hobfoll et al., 2018).

### 5.2 Practical implications

Two implications follow for private universities seeking to strengthen collegial relationships and coordination capacity.

First, professional development should prioritize relationally proximal EI competencies—emotion regulation, empathy, and social interaction skills—because these facets showed the strongest unique associations with IR. Evidence indicates that socio-emotional competencies can be developed through structured interventions, with downstream benefits for interpersonal functioning, professional adjustment, and relational quality at work (Hodzic et al., 2018; Gilar-Corbi et al., 2019; Côté, 2017; Oliveira et al., 2021). For private universities, training can be designed around (a) regulating emotions under conflict and service pressure, (b) empathic communication and perspective-taking, and (c) social skill practices such as feedback conversations, coordination routines, and relationship repair.

Second, institutions may treat administrative/extra-role assignments as developmental opportunities rather than purely instrumental workloads, because teachers with such experience showed higher EI and select facets. However, this implication must be framed dialectically: extra-role assignments can also elevate demands and deplete resources if unsupported, potentially undermining well-being and relationships (Bakker and Demerouti, 2017; Hobfoll et al., 2018). Therefore, a developmental framing should be paired with safeguards, including clear role boundaries, protected time, workload protection, mentoring, and psychologically safe climates, to ensure relational gains rather than resource loss (Frazier et al., 2017; Newman et al., 2017).

### 5.3 Limitations

Several limitations temper interpretation. First, the cross-sectional, single-source design limits causal inference and may inflate associations due to common method variance; thus, the EI–IR relationships should not be interpreted as causal effects (Podsakoff et al., 2003; Podsakoff et al., 2012). Second, EI was measured via self-report, which may reflect self-perceptions and social desirability in addition to competence; multi-method assessment would strengthen construct interpretation (MacCann et al., 2020; Mayer et al., 2016). Third, the sample was drawn from private universities in one province; replication across regions and institutional types (public vs. private; research-intensive vs. teaching-focused) is needed to clarify generalizability and boundary conditions (Zhang et al., 2021; Wilkins and Neri, 2019).

### 5.4 Future research

Future research can strengthen inference and extend mechanism understanding in four ways. First, adopt longitudinal or multi-wave designs (e.g., EI → later IR) and incorporate multi-source outcomes (peer-rated IR; supervisor-rated cooperation) to reduce method bias and clarify temporal ordering (Podsakoff et al., 2012; MacCann et al., 2020). Second, test behavioral mechanisms linking EI to IR, such as emotion regulation → conflict management → trust, or empathy → prosocial helping → mutual acceptance, using mediation designs and behavioral indicators (Côté, 2017; Hobfoll et al., 2018). Third, adopt multilevel designs with teachers nested within departments or units to examine cross-level interactions, such as whether psychological safety, leadership support, and collegial climate strengthen or weaken the association between EI and workplace interpersonal relationships (Frazier et al., 2017; Newman et al., 2017; Liu et al., 2016). Fourth, qualitative or mixed-methods follow-up studies could provide a richer understanding of how teachers with high EI navigate challenging collegial interactions in everyday academic settings. Fifth, model the developmental interplay between EI and role exposure: quasi-experimental designs around administrative role transitions (before/after taking extra-role duties) could help distinguish selection from socialization processes (Bakker and Demerouti, 2017; Hobfoll et al., 2018).

## Data Availability

The raw data supporting the conclusions of this article will be made available by the authors, without undue reservation.
